# Qualitative Enhancement of the Tooth–Filling Interface Using Cold Atmospheric Plasma

**DOI:** 10.3390/dj13090406

**Published:** 2025-09-04

**Authors:** Madline Priska Gund, Jusef Naim, Muhammad al Muhammad, Antje Lehmann, Axel Schindler, Matthias Hannig, Stefan Rupf

**Affiliations:** 1 Clinic of Operative Dentistry, Periodontology and Preventive Dentistry, Saarland University Medical Centre, Kirrberger Straße 100, Building 73, 66424 Homburg, Germany; jusef.naim@uksh.de (J.N.); matthias.hannig@uks.eu (M.H.); 2 Leibniz Institute of Surface Modification (IOM), Permoserstraße 15, 04318 Leipzig, Germany; 3 ADMEDES GmbH, Rastatter Straße 15, 75179 Pforzheim, Germany; 4 Piloto Consulting Ion Beam and Plasma Technologies, 04668 Grimma, Germany; 5 Synoptic Dentistry, Saarland University, Kirrberger Straße 100, 66421 Homburg, Germany

**Keywords:** plasma in dentistry, composites, dentin bonding agents, interfaces, cold atmospheric plasma

## Abstract

**Objective**: To evaluate the effects of cold atmospheric plasma (CAP) on adhesive bonding in Class II composite restorations in vitro. **Methods**: Forty-eight standardized Class II cavities were assigned to six groups (*n* = 8), varying in phosphoric acid conditioning, CAP treatment (1.5 W or 3 W), composite filling, and thermo-mechanical loading (TML). Evaluations included dye penetration, adhesive layer morphology, resin tag length, and hybrid layer thickness. **Results**: CAP combined with phosphoric acid (H_3_PO_4_) significantly increased hybrid layer thickness and resin tag length (*p* < 0.006). The lowest dye penetration was observed in Groups 1 and 4. **Conclusions**: CAP in combination with phosphoric acid improved the adhesive interface in Class II cavities. CAP alone showed limited benefits, and higher power levels may negatively affect bonding.

## 1. Introduction

There are many risks that can compromise and weaken the adhesive–dentin bond. For example, the incompatibility of hydrophobic photo-initiators and hydrophilic monomer composition, improper light curing, and remaining water in interfibrillar spaces of the collagen network [1]. The durability of the adhesive–dentin bond may be affected by extrinsic polymer hydrolysis and enzyme-mediated collagen degradation [2]. Discrepancies between acid etching and monomer penetration into the hybrid layer make the collagen network susceptible to further enzymatic degradation as it is exposed [1]. Furthermore, unreacted monomers may increase long-term instability [3].

Cold atmospheric plasma (CAP) is widely used in medicine and dentistry today. Among others, it is used for the treatment of oral biofilm-related infections, the optimization of implant surfaces, the treatment of viral infections like herpes simplex, and wound and skin diseases [4]. Some older and recent studies have also addressed the scientific question of the extent to which cold atmospheric plasma can improve the adhesive dentin bond [5,6,7,8].

Non-thermal argon plasma can improve the permeability of the self-etch adhesive in dentin, increase surface wettability, and clarify the smear layer, which ultimately leads to a better bond strength between dentin and the self-etch adhesive system [9].

2-Hydroxyethylmethacrylate (HEMA), a typical dental monomer, and its grafting onto dentin collagen are improved by non-thermal atmospheric plasma brush. Grafting efficacy depends on input power and the treatment time of the plasma source [10]. In addition, the initial retentive strength between a zircona crown and a titanium implant abutment using a self-adhesive resin cement can be improved by non-thermal atmospheric plasma [11], as can the initial shear bond strength between yttria-stabilized tetragonal zirconia polycrystal and self-adhesive resin cement. The achievable improvement of the initial shear bond strength depends on the selected cement [12].

A 2021 systematic review summarizes that dentin wetting is enhanced by cold atmospheric plasma, regardless of gas phase and exposure time, when comparing self-etch and total-etch protocols. In addition, short- and long-term effects on bond strength were observed compared to the controls. For all these observations, an exposure time of 30 s and a distance of 10 mm between the plasma source and the surface were sufficient to obtain positive effects of helium and argon plasma [1].

Since there are no differences in the treatment of class II cavities by direct and indirect resin composite restorations and minimally invasive dental medicine is the future, the longevity and stability of the dentin adhesive bond is all the more important [13].

Thermo-mechanical aging is widely used in dentistry to perform in vitro investigations to assess the quality of restoration materials. In particular, the ability to simulate and compare the fatigue of the dentin–composite interaction zone promises to provide important information for this study. It is expected that CAP will influence and possibly improve the interaction through its effect on the dentin.

To the best of the author’s knowledge, this is the first study to investigate the effects of cold atmospheric plasma on adhesive–dentin bonding of three-surface standard class II cavities. Furthermore, for the first time, molars conditioned with a 37% phosphoric acid gel and helium plasma or with helium plasma alone were subjected to artificial aging alternating between thermal and mechanical loading. Thermo-mechanical aging is widely used in dentistry to perform in vitro investigations to assess the quality of restoration materials. In particular, the ability to simulate and compare the fatigue of the dentin–composite interaction zone promises to provide important information for this study. It is expected that CAP will influence and possibly improve the interaction through its effect on the dentin. The adhesive layer of the tooth–composite interaction zone is still a critical area of restoration. Methods to improve tooth–composite interactions by stabilizing the adhesive layer would increase the durability of the restorations or at least reduce the discoloration in the marginal area.

## 2. Material and Methods

### 2.1. CAP Source

A plasma source developed at the Leibniz Institute for Surface Modification in Leipzig and already described in the literature was used [14,15]. The plasma was generated by microwave excitation (2.45 GHz) with the mean electric power input being 1.5 W or 3 W. The microwave pulse had a width of 3 µs and an output of 300 W. Helium (3.5 L/min) was used as carrier gas. The plasma source was mounted on a computer-controlled 3-axis motion system in order to ensure reproducible parameters of time, distance, and scanning mode.

### 2.2. Plasma Treatment Parameters

Treatment was performed at cavities of groups 2 to 6 (Table 1) at a distance of 3.5 mm between the plasma source and cavity bottom with a line speed of 5.5 mm/s. The feed from line to line was 0.1 mm, resulting in a surface-related plasma treatment of 1.82 s/mm^2^. Following treatment of the occlusal cavity share, the tooth was rotated at a 90° angle in order to treat both proximal cavities. The parameters were adjusted by means of infrared thermography (Optris PU, Optris GmbH, Berlin, Germany) in such a way that a temperature of 32 °C was achieved at the impact point at a mean electric input of 1.5 W. When the input increased to 3 W, the temperature at impact point increased to 40 °C.

### 2.3. Cavities

Three-surface standard class II cavities were prepared in 48 freshly extracted, caries-free human molars (lower first and second molar), which had been stored in 0.1% thymol solution. The cavities had the following dimensions: oral vestibular in the occlusal region: 3 mm; oral vestibular at the proximal shoulders: 4 mm; width of the proximal shoulders: 1.2 mm; occlusal depth: 2 mm; and occlusal-cervical depth: 4 mm. Preparation was performed with a rounded preparation diamond of 100 µm grain (pear-shaped, 010, Intensiv^®^, Viganello, Switzerland) and finished with a diamond burr of the same shape and grain of 25 µm (pear-shaped, 010, Intensiv^®^, Viganello, Switzerland). After cavity preparation, the 48 molars were randomly allocated to 6 groups (*n* = 8 teeth). As this is a pilot study, no sample size calculation was carried out.

### 2.4. Treatment of Cavities and Group Disposition

The cavities were conditioned with a 37% phosphoric acid gel or plasma jet alone (mean electric input 3 W) and in combinations (3 W or 1.5 W and phosphoric acid; categorization of groups: Table 1). Cavities of groups 1 to 5 were conditioned with 37% phosphoric acid gel, starting at the enamel areas for a total of 30 s and 10 s in the dentinal areas. Afterwards, all cavities were filled with composite, using the adhesive system OptiBond FL and the composite Herculite XRV (both Kerr, Biberach, Germany) according to the manufacturer’s instructions. A polymerization lamp (Astralis 10, Ivoclar Vivadent, Schaan, Liechtenstein) was used for light curing for 20 s, and the incremental technique was used to apply the filling material. To simulate clinical stress, teeth were subjected to artificial aging, alternating between thermal and mechanical loading. Of the six groups, five were subjected to thermomechanical stress (G 2-6) (Table 1). Groups 1 and 6 served as control groups.

### 2.5. Preparation for Examination

The root apices of the teeth were sealed with sticky wax, and all surfaces were coated with two layers of nail varnish from beyond 1 mm of the gingival margin of the restorations. The teeth were soaked in 0.1% methylene blue dye in a 37 °C water bath for 24 h.

After removal from the dye solution, they were thoroughly washed under tap water, sectioned mesiodistally into halves along their long axis using a diamond disc with water coolant (Conrad Apparatebau GmbH, Clausthal-Zellerfeld, Germany), and polished (Metkon Instruments Inc., Osmangazi/Bursa, Turkey). Dye penetration along the filling margin was documented with a digital photo camera (Canon EOS 400d, Canon Deutschland GmbH, Krefeld, Germany) on a photo reproduction stand (Copylizer eVision exe.cutive, Kaiser Fototechnik GmbH und Co. KG, Buchen, Germany). The depth of dye penetration was evaluated with a scoring system (Table 2).

### 2.6. Statistical Analysis

The scores of dye penetration observed in the groups were checked with the Mann–Whitney-U test for differences. Group 2 was used as a reference for comparison. Values *p* < 0.05 were considered statistically significant.

The dentin–composite interaction zone was evaluated according to the following criteria: the visibility of the adhesive/hybrid layer (%), thickness of the adhesive/hybrid layer (µm), length of intratubular penetration (tags, µm), and occurrence of peritubular penetration. Ten images each, spread over the tooth–composite interaction zone of the cavity floor, were evaluated for every sample. Mean values were calculated from the measured values for the thickness of the adhesive/hybrid layer and length of intratubular penetration. These means were compared by t test for independent samples. All tested groups were compared with group 2 (significant level *p* < 0.05).

## 3. Results

### 3.1. Dye Penetration

None of the groups investigated had an exclusive score of 0 in the dye penetration test (Table 2 and Table 3). The highest shares of dye-free tooth–composite interaction zones were observed in group 1 and 4. Plasma treatment with 3 W input after phosphoric acid conditioning (G 3) and plasma treatment alone (G 6) showed higher dye penetration rates.

### 3.2. Scanning Electron Microscopic Analysis

#### 3.2.1. Overview

**Figure 1:**
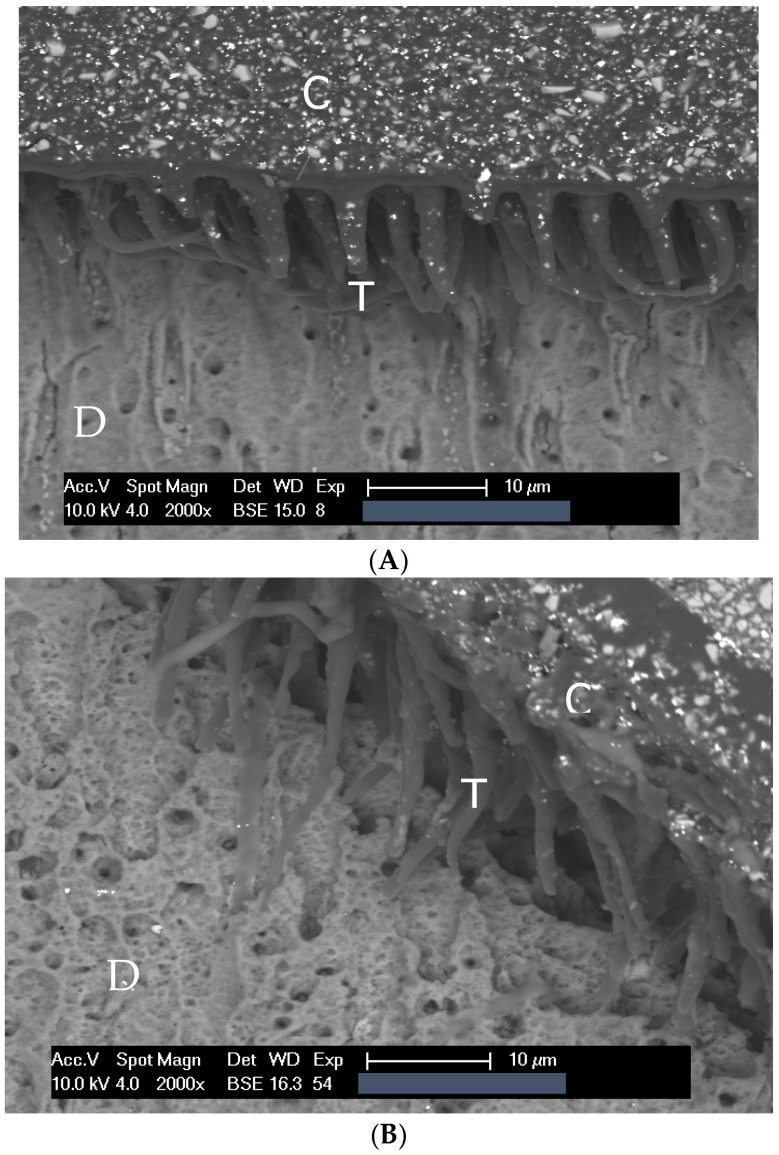

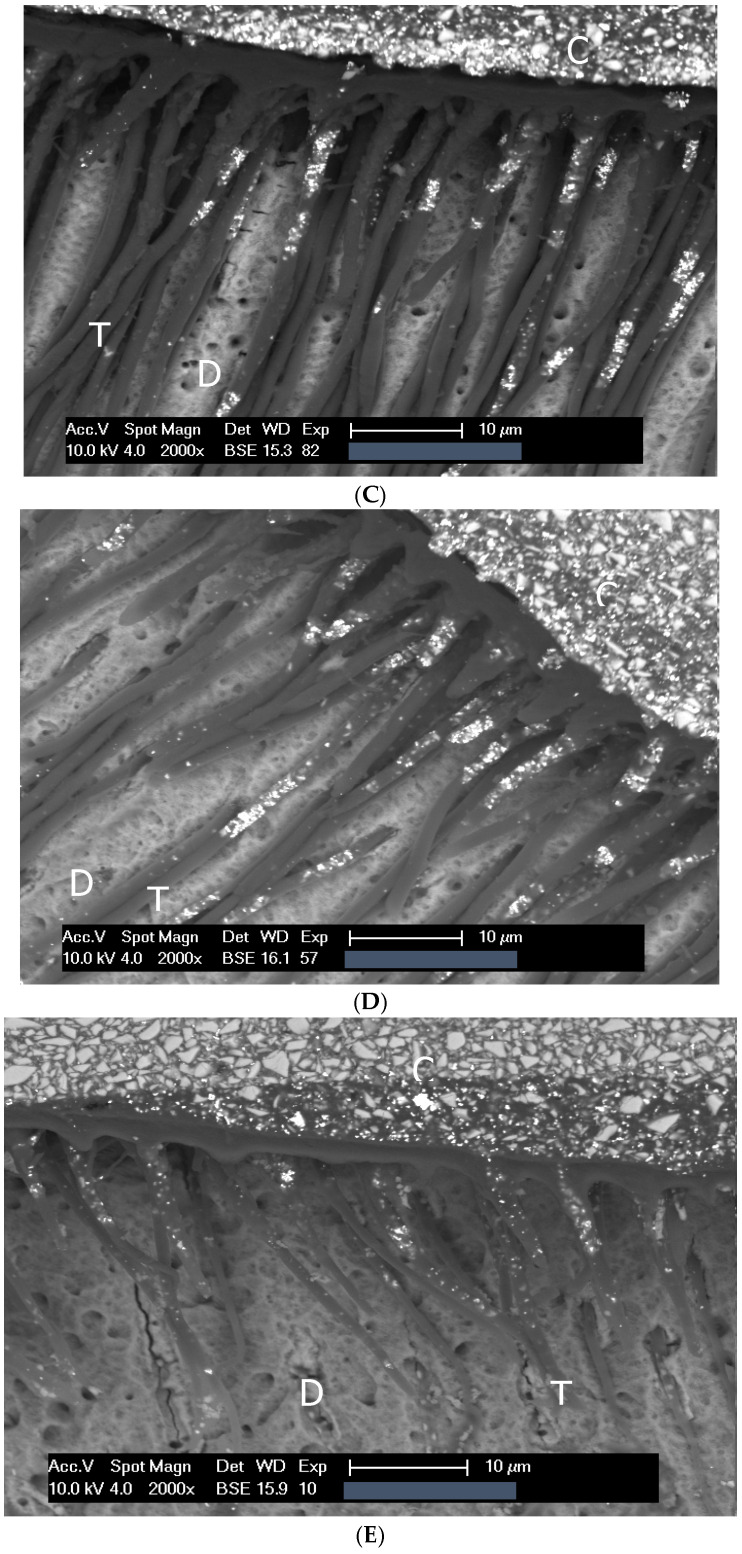

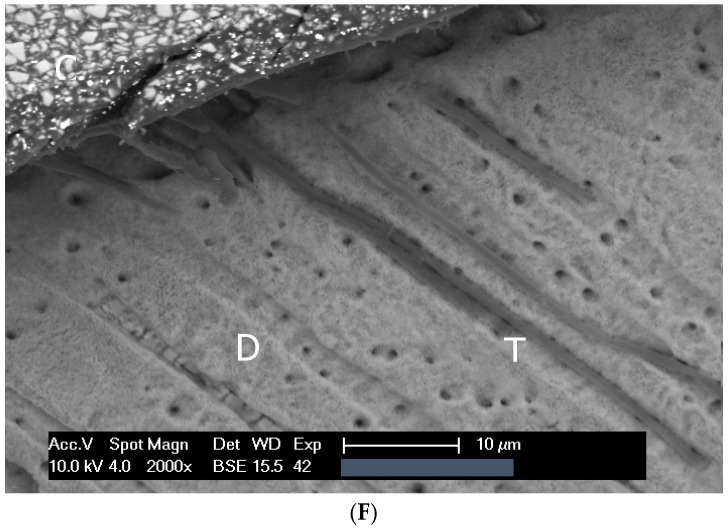
SEM images of the interaction zone for the groups (**A**) G1: E-F, (**B**) G2: E-F-TB, (**C**) G3: E-P3W-F-TB, (**D**) G4: E-P1.5W-F-TB, (**E**) G5: P3W-E-F-TB, and (**F**) G6: P3W-F-TB. Markings within the images; C: composite, T: resin tags, and D: dentin.

**Figure 2:**
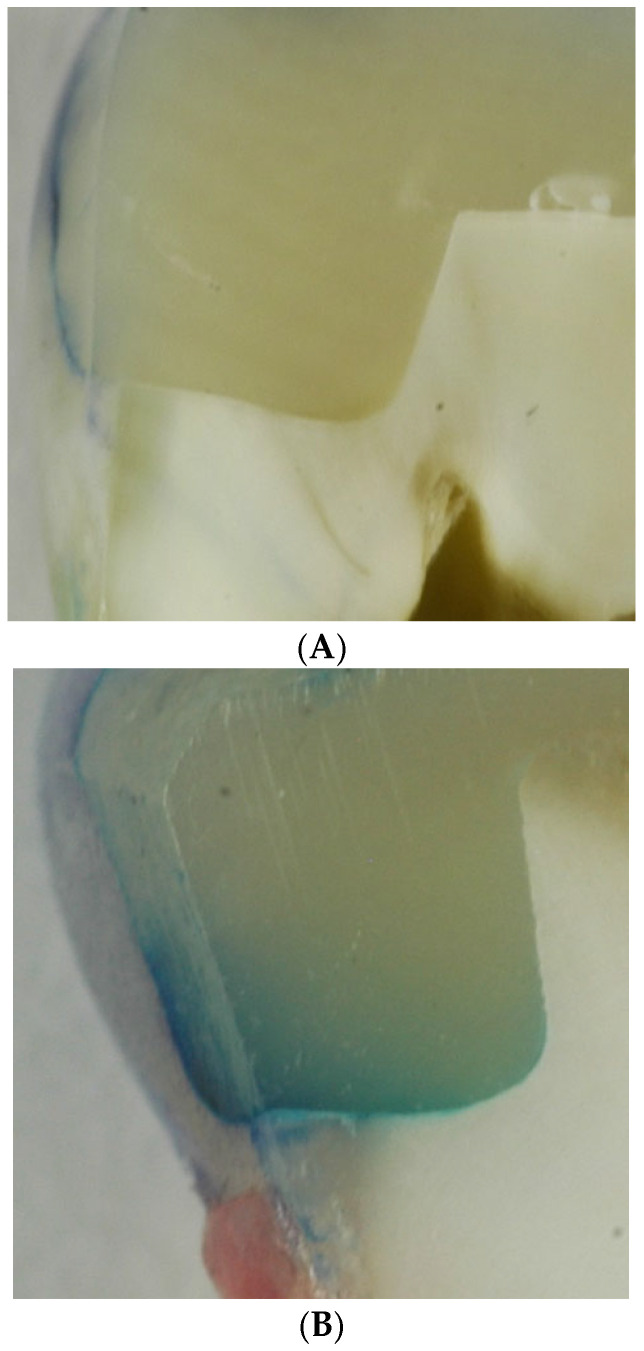

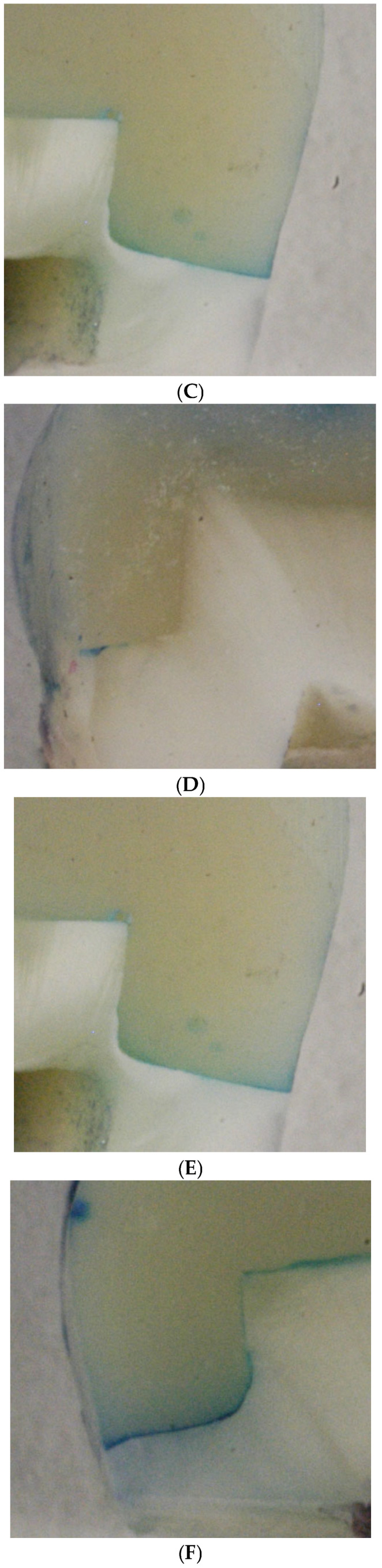
Light-microscopic images of the dye penetration (blue stain) into the interaction zone for the groups (**A**) G1: E-F, (**B**) G2: E-F-TB, (**C**) G3: E-P3W-F-TB, (**D**) G4: E-P1.5W-F-TB, (**E**) G5: P3W-E-F-TB, and (**F**) G6: P3W-F-TB.

Independent of thermomechanical stress and the form of surface conditioning, the structures of dentin, the interaction zone, and the composite were recognizable in all the investigated objects. In comparison with the surface exclusively conditioned with phosphoric acid (G 1 and 2), the interaction zones in the three groups additionally treated with plasma (G 3, 4, and 5) appeared wider, and adhesive penetration was increased. Objects that were treated with plasma (G 6) but without phosphoric acid tended to show shorter adhesive tags. Representative SEM and light microscopic images are provided in Figure 1 and Figure 2A–F.

The hybrid layer was detectable in all the investigated objects. Thermomechanical stress led to a weakening of the tooth–composite interaction zone. While only a small share of crack formations was detected in SEM imaging in group 1 (the control group without stress), these occurred regularly in all groups subjected to stress.

#### 3.2.2. Aging of Fillings

Significant aging of the fillings was observed in Group 2 after a total of 3000 cycles of thermal load in a water bath (5 °C and 55 °C) and 300,000 cycles of mechanical loading in a mechanical pressure simulator with a vertical force of 50 N in a frequency of 1.8 Hz.

#### 3.2.3. Hybrid Layer

The mean hybrid layer presented showed significant differences between the various groups. The combined application of phosphoric acid and plasma, regardless of the order of application, results in a stronger hybrid layer.

(G 2 vs. 3, 4, and 5, *p* = 0.001; 0.001; and 0.006, respectively). Conditioning with plasma alone did not significantly reduce the thickness of the hybrid layer. Thermomechanical stress did not have an influence on the hybrid layer’s thickness (comparison G 1 and 2, *p* = 0.56).

#### 3.2.4. Adhesive Tags

Intratubular and peritubular adhesive penetration, expressed in the length of the adhesive tags in the dentin tubules and their branching, was influenced by plasma treatment. Significantly longer mean tag lengths were observed in those groups conditioned by phosphoric acid and plasma treatment (G 2 vs. 3, 4, and 5, *p* = 0.002; 0.002; and 0.001, respectively). The rate of occurrence of lateral adhesive penetrations also increased (Table 3). The application of plasma treatment alone tended to shorten adhesive tags (G 2 vs. 6, *p* = 0.08). Thermomechanical aging did not have an influence on the length and occurrence of intratubular adhesive tags (*p* = 0.21) (Table 3).

## 4. Discussion

To the best of the author’s knowledge, this is the first study to investigate the effects of cold atmospheric plasma on adhesive–dentin bonding of three-surface standard class II cavities. Furthermore, for the first time, molars conditioned with a 37% phosphoric acid gel and CAP or with CAP alone were subjected to artificial aging alternating between thermal and mechanical loading. For this study, a model based on extracted teeth was used to enable conclusions to be drawn for real cavities. The composite fillings placed in this study were subjected to thermomechanical loading to evaluate possible morphological changes in the tooth–composite interaction zone due to plasma treatment.

Some studies have already looked at how plasma treatment can improve the tooth–composite interface. However, the studies were on dentin discs, not cavities. In addition, thermocycling and water storage were partially carried out, but no thermomechanical loading was carried out to age the adhesive–dentin bonding [5,8,16,17,18].

The methods were chosen to show differences between the test and control groups. The stress parameters were adjusted to show damage in the filling margins in the thermomechanical stress control group. The stabilization of the tooth–composite interaction zone by plasma treatment had to lead to a lessening of the defects in the interaction zone, which also means a weakening of the interaction zone would result in a more pronounced failure rate of the fillings. Finally, we chose parameters for plasma treatment that can be used in dental practice. Thus, the temperature was set to values of 40 °C and 32 °C for one parameter, respectively. Also, with regard to the duration of treatment time, an adjunct method appropriate for cavity treatment was chosen with 1.8 s/mm^2^. Typical cavities have sizes of 1 mm^2^ up to approx. 30 mm^2^, thus requiring treatment times of a few seconds up to one minute.

The exposure time has often been discussed in the literature in order to improve the bond strength [6,7,17,19].

Basically, plasma treatment up to 100 s showed an increase in the interfacial bonding strength, while a prolonged one (e.g., 5 min) led to a decrease [6]. Other authors generally confirm better bond strength for plasma treatment [5,16,17,20,21].

Han et al. found out that thermocycling did not decrease the microtensile bond strength (MTBS) of resin composite to dentin in the control or conventional plasma group but increased in the pulsed plasma group. He also could show that plasma treatment improved the MTBS compared with the control group. He could not show significant differences between the plasma treatment groups. The study investigated the effect of low-power non-thermal atmospheric pressure plasma (NT-APP) treatments in pulsed and conventional modes [22]. Hirata et al. investigated, in 2016, the influence of atmospheric pressure plasma (APP) treatment on the microtensile dentin bond strength of two etch-and-rinse adhesive systems after one week and one year of water storage. He additionally observed the micromorphology of resin-dentin interfaces under scanning electronic microscopy (SEM). Dentin bond strength increased for XP Bond at one week, but no effects could be observed for Optibond FL, which was used in our study. The effect was not stable after one year for XP Bond. The bond strength of Optibond FL was not influenced at all [5]. The authors are not aware of any other studies that have examined the effects of plasma treatment on the MTBS of Optibond FL.

Investigating the effects of plasma treatment on the tooth and composite showed that it affects how dye penetrates and thus the margins of the filling at the gum. At a low electrical input of 1.5 W with phosphoric acid etching, dye penetration decreased compared to the control group. However, in the groups where a higher electrical input of 3 W was used, the dye penetration increased. This confirms the results of the previous studies, which showed that excessive plasma input can harm the interaction zone. Cold plasma at high performance can destroy free collagen and is an etching process; on the other hand, this effect is attributed to an over-etching of the dentin surface. The authors are not aware of any other studies that have also investigated dye penetration in connection with the margins of the fillings.

The plasma jet and phosphoric acid conditioning affected the interaction zone between tooth and composite. The components of the interaction zone were visible in all specimens. Plasma treatment did not affect the components. Thermomechanical stress weakened the adhesive layer, which led to cracking in the interaction zone. In addition to cracking during adhesive and composite processing, the interaction zone is also stressed during specimen preparation and SEM analysis. This can lead to cracking. Vacuum and electron bombardment play an important role. These artifacts form in an uncontrolled manner. However, it can be concluded that the presence or absence of artifacts is a sign of differences in the stability of the interaction zone.

In this study, cracks in the interaction zone occurred more often in thermo-mechanically loaded groups. In contrast, non-loaded fillings showed this phenomenon only in rare cases. When comparing aged controls and cavities treated with atmospheric plasma, no differences were observed. This suggests that plasma treatment does not strengthen the interaction zone between tooth and composite and does not reduce the difference between stressed and unstressed specimens.

These results are confirmed by a study conducted by Ayres et al. [23]. They investigated the bonding effect of a multimode adhesive (Scotchbond Unviversal in self-etch or etch-and-rinse mode) on controls and plasma-treated dentin using a mini-interfacial fracture toughness test (mini-iFT). The mini-iFT did not change in any group after 6 months of ageing (6 months of storage in distilled water). However, thermomechanical stress was not carried out. Therefore, the results are only comparable to a limited extent [23].

The unstressed control group and thermo-mechanically stressed control fillings without plasma treatment had the same adhesive/hybrid layer thickness. Plasma treatment had a significant effect. Plasma treatment did not significantly reduce the hybrid layer thickness. However, when phosphoric acid conditioning and plasma treatment were combined, the hybrid layer thickness increased significantly.

Both plasma treatment and phosphoric acid conditioning are etching processes in which mineralized basic substances are dissolved out of the dentin surface. Deeper demineralization in the dentin should therefore result in a thicker hybrid layer and thus increase the thickness of the overall tooth–composite interaction zone. The highest values for hybrid layer thickness were obtained for conditioning with phosphoric acid followed by plasma treatment at low power (G 4). It is possible that the collagen network is denatured during plasma treatment at about 40 °C. At lower electrical power with resulting temperatures of 32 °C, the collagen is not denatured but only made more susceptible to penetration by the agglomeration of reactive oxygen groups [6]. Since the primer substance of the adhesive system used is a mild acid and therefore hydrophilic, the susceptibility to penetration might have been improved by increased wettability after plasma treatment. The current literature confirms the findings of this study. Stasic et al. describe better adhesive penetration on the dentin surface compared to the controls due to increased dentin wetting and surface free energy [24]. Ayres et al. lead the improved microtensile bond strength, possibly to increase in nanohardness and the Young’s modulus of the hybrid layer. Furthermore, to better adhesive infiltration, since dentin hydrophilicity could be improved [7]. Dong et al. describe that a non-thermal argon plasma treatment can improve the penetration of adhesive monomers due to the prior dissolution of the collagen structure. Consequently, a thicker hybrid layer and longer formation of resin tags can be expected, which improve adhesive interfacial adhesion [25]. In another study, Dong et al. confirmed their findings [20,26]. The advantages of plasma treatment in terms of better penetration of the adhesive, improvement of mechanical adhesion, a thicker hybrid layer, longer formation of resign tags and the polymerization rate, better bond strength, and finally long-term durability have been widely studied and demonstrated in the current literature [7,16,17,25,27,28].

## 5. Conclusions

This study shows for the first time that CAP can improve the tooth–composite interaction in three-surface standard class II cavities by increasing resin penetration. Plasma treatment without acid phosphoring was less effective than when used with acid phosphoring. CAP does not increase the resistance of the interaction zone between tooth and composite to mechanical influences and therefore does not minimize the difference between stressed and unstressed specimens. Limitations regarding higher plasma powers were detected.

## Figures and Tables

**Table 1:** Experimental groups and processing parameters. Each group contains 8 cavities. Abbreviations: E: phosphoric acid conditioning, F: filling, TB: thermomechanical alternating load; P3: plasma treatment 3 W input power; and P1.5: plasma treatment 1.5 W input power.

Groups	Conditioning (Phosphoric Acid)	Plasma Treatment	Filling	Thermo-Mechanical Loading
G1: E-F	Enamel 30 s Dentin 10 s, Water spray 15 s	Non	Optibond FL/Herculite XRV	non
G2: E-F-TB	Enamel 30 s Dentin 10 s, Water spray 15 s	Non		3 × (1000 cycles of thermal load and 100,000 cycles mechanical stressing)
G3: E-P3W-F-TB	Enamel 30 s Dentin 10 s, Water spray 15 s	3 W, 1.8 s/mm^2^, 40 °C		
G4: E-P1.5W-F-TB	Enamel 30 s Dentin 10 s, Water spray 15 s	1.5 W, 1.8 s/mm^2^, 40 °C		
G5: P3W-E-F-TB	Enamel 30 s Dentin 10 s, Water spray 15 s	3 W, 1.8 s/mm^2^, 40 °C		
G6: P3W-F-TB	Non	3 W, 1.8 s/mm^2^, 40 °C		

**Table 2:** Scoring for the cervical mikroleakage.

**Score 0**	No dye penetration
**Score 1**	Dye penetration limited to enamel
**Score 2**	Dye penetration beyond the dentino-enamel junction but limited to 2/3rds of the cervical wall length
**Score 3**	Dye penetration beyond 2/3rds of the cervical wall length but not to the pulpal wall
**Score 4**	Dye penetration to the pulpal wall

**Table 3:** Adhesive layer morphology in dentin: formation of adhesive layer, intra-tubular (tags, µm), lateral adhesive penetration (lateral branches present, %), thickness hybrid layer (µm), and dye penetration (percentage of Score 0, average score).

Group	Hybrid Layer	Dye Penetration, % Score 0	Average Dye Penetration Score	Average Thickness Hybrid Layer	Average Intratubular Adhesive Penetration	Lateral Branches (%)
G1: E-F	100%	75%	0.875	2.66 µm	36.16 µm	37.5%
G2: E-F-TB	100%	44%	1.125	2.41 µm	31.73 µm	37.5%
G3: E-P3W-F-TB	100%	13%	3.125	3.1 µm *	60.27 µm *	87.5%
G4: E-P1.5W-F-TB	100%	81%	0.5	3.94 µm *	74.44 µm *	87.5%
G5: P3W-E-F-TB	100%	44%	1.125	3.11 µm *	55.83 µm *	75%
G6: P3W-F-TB	100%	6%	2.875	1.83 µm	25.21 µm	25%

* statistically significant difference in comparison to group 2 (standard procedure, control).

## Data Availability

The dataset used and analyzed during the current study is available from the corresponding author upon reasonable request.
